# High Voltage and Train-Surfing Injuries: A 30-Year Retrospective Analysis of High-Voltage Trauma and Its Impact on Cardiac Biomarkers

**DOI:** 10.3390/jcm14144969

**Published:** 2025-07-14

**Authors:** Viktoria Koenig, Maximilian Monai, Alexandra Christ, Marita Windpassinger, Gerald C. Ihra, Alexandra Fochtmann-Frana, Julian Joestl

**Affiliations:** 1 Division of Plastic, Aesthetic and Reconstructive Surgery, Medical University of Vienna, Waehringer Guertel 18–20, 1090 Vienna, Austria; viktoria.koenig@meduniwien.ac.at (V.K.); n11927646@students.meduniwien.ac.at (M.M.); alexandra.christ@meduniwien.ac.at (A.C.); alexandra.fochtmann@meduniwien.ac.at (A.F.-F.); 2 Department of Anesthesia, Critical Care and Pain Medicine, Division of General Anesthesia and Intensive Care Medicine, Medical University Vienna, 1090 Vienna, Austria; marita.windpassinger@meduniwien.ac.at (M.W.); gerald.ihra@meduniwien.ac.at (G.C.I.); 3 Private Clinic, Spitalgasse 19, 1090 Vienna, Austria

**Keywords:** high-voltage electrical injury, train surfing, cardiac biomarkers, burn trauma, adolescent risk behavior, intensive care, electrocardiography, creatine kinase, troponin, mortality, injury prevention

## Abstract

**Background**: High-voltage electrical injuries (HVEIs) represent a complex and life-threatening entity, frequently involving multi-organ damage. While traditionally linked to occupational hazards, train surfing—riding on moving trains—and train climbing—scaling stationary carriages—have emerged as increasingly common causes among adolescents. Popularized via social media, these behaviors expose individuals to the invisible danger of electric arcs from 15,000-volt railway lines, often resulting in extensive burns, cardiac complications, and severe trauma. This study presents a 30-year retrospective analysis comparing cardiac biomarkers and clinical outcomes in train-surfing injuries versus work-related HVEIs. **Methods**: All patients with confirmed high-voltage injury (≥1000 volts) admitted to a Level 1 burn center between 1994 and 2024 were retrospectively analyzed. Exclusion criteria comprised low-voltage trauma, suicide, incomplete records, and external treatment. Clinical and laboratory parameters—including total body surface area (TBSA), Abbreviated Burn Severity Index (ABSI), electrocardiogram (ECG) findings, intensive care unit (ICU) and hospital stay, mortality, and cardiac biomarkers (creatine kinase [CK], CK-MB, lactate dehydrogenase [LDH], aspartate transaminase [AST], troponin, and myoglobin)—were compared between the two cohorts. **Results**: Of 81 patients, 24 sustained train-surfing injuries and 57 were injured in occupational settings. Train surfers were significantly younger (mean 16.7 vs. 35.2 years, *p* = 0.008), presented with greater TBSA (49.9% vs. 17.9%, *p* = 0.008), higher ABSI scores (7.3 vs. 5.1, *p* = 0.008), longer ICU stays (53 vs. 17 days, *p* = 0.008), and higher mortality (20.8% vs. 3.5%). ECG abnormalities were observed in 51% of all cases, without significant group differences. However, all cardiac biomarkers were significantly elevated in train-surfing injuries at both 72 h and 10 days post-injury (*p* < 0.05), suggesting more pronounced cardiac and muscular damage. **Conclusions**: Train-surfing-related high-voltage injuries are associated with markedly more severe systemic and cardiac complications than occupational HVEIs. The significant biomarker elevation and critical care demands highlight the urgent need for targeted prevention, public awareness, and early cardiac monitoring in this high-risk adolescent population.

## 1. Introduction

High-voltage electrical injuries represent one of the most complex and devastating forms of trauma, often leading to multisystem involvement and significant morbidity [1,2,3,4,5,6,7]. While traditionally associated with occupational accidents, an alarming rise in non-occupational causes—particularly among adolescents and young adults—has emerged in recent years [1]. Among them, train surfing, a high-risk behavior involving riding on the exterior of moving trains, has become an increasingly recognized cause of high-voltage trauma [8,9,10]. Closely related is train climbing, defined as the act of scaling stationary train carriages [1]. Both behaviors are propagated via social media platforms and are often perceived as acts of thrill-seeking or rebellion, with participants typically unaware of the severe dangers involved [2,11,12,13].

A key risk lies in the invisible and potentially lethal electric arc, which can transmit thousands of volts through the air even in the absence of direct contact with overhead power lines—typically charged with 15,000 volts [5,6,11,14]. Contact or proximity to these lines may result in catastrophic injuries, including third-degree burns, arrhythmias, cardiac arrest, rhabdomyolysis, and severe secondary trauma due to falls or collisions with bridges and other infrastructure [6,15,16,17,18,19]. Particularly in train surfing, cranial entry points are common, resulting in a vertical current pathway and disproportionately severe systemic outcomes [4].

Compared to occupational high-voltage injuries—which more often affect the extremities and occur in a regulated environment with safety precautions—train-surfing injuries tend to be more extensive, occur in younger patients, and present greater surgical and medical complexity [2,4]. Despite this, train surfing remains understudied in the literature on electrical trauma [8,11,20,21,22].

The present study provides a 30-year retrospective analysis of high-voltage injuries treated at a Level 1 burn center in Austria, comparing train-surfing cases with those sustained in work-related settings. With a specific focus on cardiovascular outcomes, this study investigates the frequency and severity of pathological ECG findings and elevations in cardiac biomarkers such as creatine kinase (CK), troponin, myoglobin, and CK-MB. Burn severity was additionally assessed using total body surface area (TBSA) and the Abbreviated Burn Severity Index (ABSI), a validated scoring system that estimates burn mortality risk based on factors such as age, TBSA, presence of full-thickness burns, inhalation injury, and gender. By identifying group-specific differences, the study seeks to contribute to both clinical understanding and the development of targeted prevention strategies addressing this emerging and particularly dangerous injury mechanism.

## 2. Materials and Methods

Following ethical approval by the Ethics Committee of the Medical University of Vienna (Approval No. 1384/2023), we conducted a retrospective analysis including all patients with confirmed high-voltage electrical injuries admitted to the Department of Plastic, Reconstructive and Aesthetic Surgery at the Vienna General Hospital between January 1994 and December 2024. Data were retrieved from the hospital’s electronic documentation systems (AKIM and PDMS).

Patients were included if they sustained electrical trauma from a source ≥ 1000 volts, either through occupational exposure or train surfing. Exclusion criteria comprised low-voltage injuries (<1000 V), non-electrical burns, cases prior to 1994, treatment in external hospitals, suicide attempts, and incomplete records. After applying these criteria, 81 patients were included in the final cohort.

The following parameters were analyzed: demographic data (age, sex), injury mechanisms (train-surfing or work-related), total body surface area burned (TBSA), Abbreviated Burn Severity Index (ABSI) score, duration of ICU and total hospital stay, mortality, cardiac complications (e.g., arrhythmias, cardiac arrest, resuscitation), and laboratory parameters including creatine kinase (CK; ref. < 142 U/L), lactate dehydrogenase (LDH; ref. < 240 U/L), aspartate aminotransferase (AST; ref. < 44 U/L), troponin (ref. < 14 ng/L), myoglobin (ref. < 72 ng/mL), and creatine kinase-MB (CK-MB; ref. < 25 U/L). Electrocardiographic abnormalities such as sinus tachycardia, ST elevations, bradycardia, conduction blocks, ventricular fibrillation, and T-wave changes were recorded.

### 2.1. Standard Clinical Management

The standard protocols for high-voltage electrical injuries at our Level 1 burn center involve initial fluid resuscitation guided by urinary output, hematocrit, and serum lactate concentrations. Management of myoglobinuria includes diuretics to maintain alkaline urinary pH, alongside central venous lines and continuous hemodynamic and respiratory monitoring. Over time, strategies evolved towards individualized approaches using balanced solutions (crystalloids, albumin, high-dose vitamin C) in minimal effective volumes. Dynamic monitoring techniques (e.g., transpulmonary thermodilution, PiCCO) and bedside echocardiography guide fluid therapy. Circulatory support includes catecholamines, integrated within comprehensive ICU protocols that also encompass oxygen therapy, temperature management, and multimodal analgesia. Imaging (ultrasound, CT) was performed following stabilization, with ENT and ophthalmology consultations for suspected head or facial burns. Decompression procedures (fasciotomy, carpal tunnel release) were performed within 24 h if indicated. Full-thickness burns were treated with early excision and split-thickness skin grafting or flap reconstruction.

### 2.2. Statistical Analysis

Statistical analyses were performed using SPSS version 30.0.0.0 and R version 3.1.1. Categorical variables were analyzed with chi-square or Fisher’s exact test. Metric variables were compared with the Mann–Whitney *U* test and are presented as median [min–max], given non-normal distribution. Correlations between injury type and complications were assessed via cross-tabulation. Statistical significance was set at *p* < 0.05. Biomarkers were compared between groups on day 3 (acute phase) and day 10 (subacute phase), and paired comparisons assessed temporal changes. Planned multivariate logistic regression could not be completed due to perfect separation, where extreme biomarker elevations occurred exclusively in deceased patients.

## 3. Results

Of the 81 patients analyzed, 57 (70.37%) sustained work-related injuries, while 24 (29.63%) were due to train surfing, yielding a ratio of 2.38:1. The mean age of the total cohort was 29.73 years (range 13–73 years), with a median of 26 years (IQR 18–39). Train surfers were significantly younger, with a mean of 16.67 years and median 17 years (IQR 15–18), compared to work-related injuries with a mean of 35.23 years and median 34 years (IQR 25–42.5) (*p* = 0.008). Gender distribution was overwhelmingly male (79 males, 2 females). One female patient sustained injuries from train surfing and another in an occupational incident.

TBSA was significantly higher among train surfers (mean 49.92%, median 50% [IQR 40–59.5]) than work-related injuries (mean 17.91%, median 8% [IQR 5–25]) (*p* = 0.008). Mean ABSI score across all patients was 5.73 (median 6, IQR 4–7), with train surfers exhibiting higher scores (mean 7.29, median 7, IQR 6–8) than work-related patients (mean 5.11, median 4, IQR 4–6) (*p* = 0.008).

Hospital stay averaged 41 days (median 27, IQR 11–54), longer for train surfers (mean 66 days, median 44 [IQR 22–78]) than for work-related injuries (mean 31 days, median 19 [IQR 9–45], *p* = 0.104). ICU stay was significantly longer for train surfers (mean 53 days) compared to work-related injuries (mean 17 days, *p* = 0.008). Overall mortality was 8.6% (7/81), higher among train surfers (20.8%) than work-related cases (3.5%, *p* = 0.022).

### 3.1. Electrocardiographic Findings

Among 78 patients with complete ECG data, 40 (51%) showed pathological findings, primarily within the first 24 h post-injury. Pathological ECGs were detected in 13 train surfers (56.5%) and 27 work-related patients (49.1%) (*p* = 0.549). Sinus tachycardia, bradycardia, atrial fibrillation, and conduction disturbances were most frequent.

### 3.2. Cardiac Arrest

Cardiac arrest occurred in 11 patients (13.6%), with a higher rate in train surfers (6/24; 25%) than in work-related patients (5/57; 8.8%, *p* = 0.069). Of six arrests among train surfers, four were fatal; among work-related injuries, two fatalities followed cardiac arrest.

### 3.3. Cardiac Enzymes

To evaluate the extent of cardiac involvement following high-voltage trauma, the following laboratory parameters were assessed: creatine kinase (CK; reference < 142 U/L), lactate dehydrogenase (LDH; reference < 240 U/L), aspartate aminotransferase (AST; reference < 44 U/L), troponin (reference < 14 ng/L), myoglobin (reference < 72 ng/mL), and creatine kinase-MB (CK-MB; reference < 25 U/L). These biomarkers were measured on day 3 and day 10 after injury in both patient groups.

Cardiac biomarker levels were significantly higher in train surfers compared to patients with work-related high-voltage injuries at both time points, indicating a greater degree of cardiac stress and systemic tissue damage in the train-surfing cohort (Table 1 and Table 2).

### 3.4. Creatine Kinase

Analysis demonstrated a significant difference in CK levels between the two groups within the first 72 h post-injury (*p* = 0.042). Train surfers exhibited a markedly higher median CK value of 9672.5 U/L, compared to 263 U/L in the work-related injury group. The interquartile range (IQR) among train surfers was 5205.75 U/L, substantially broader than the IQR of 36 U/L observed in the work-related group (*p* = 0.042), reflecting greater variability in muscle injury severity.

**Figure 1:**
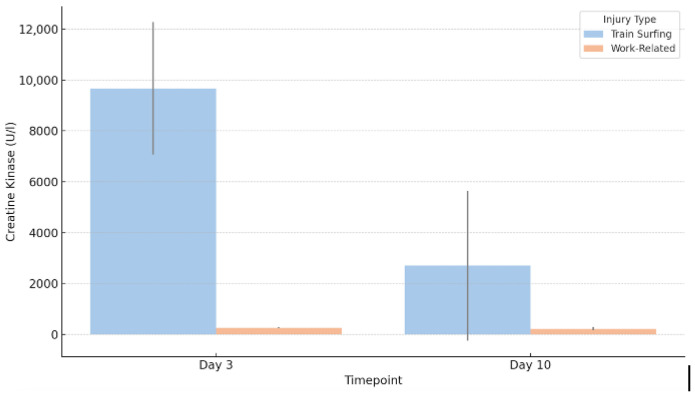
Median CK levels on day 3 and day 10.

Extreme outliers were present in both groups, with peak values reaching 162,000 U/L and 76,330 U/L, respectively. While CK levels declined over the subsequent 10-day period in both cohorts, a significant difference persisted. The train-surfing group maintained a median CK level of 2702.25 U/L (IQR: 5892.12 U/L), in contrast to a median of 221.5 U/L (IQR: 120.25 U/L) in the occupational injury group (*p* = 0.035) (Figure 1).

### 3.5. Lactate Dehydrogenase (LDH)

A comparison of LDH values within the first 72 h post-injury showed significantly elevated levels in the train-surfing group compared to work-related accidents (*p* = 0.042). The median LDH level among train surfers was 612 U/L, more than three times higher than the 182 U/L observed in the occupational cohort (*p* = 0.042). The interquartile range (IQR) also reflected this disparity, measuring 239.75 U/L in the train-surfing group versus 29 U/L in work-related cases (*p* = 0.042), indicating greater variability and more severe tissue damage. Outliers were present in both groups, with maximum values reaching 3352 U/L among train surfers and 2221 U/L in the work-related group.

**Figure 2:**
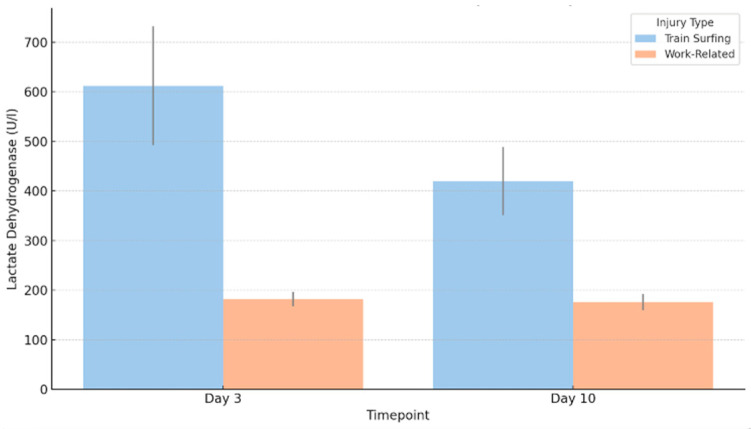
Median LDH levels on day 3 and day 10.

At day 10, LDH levels remained significantly different between both groups (*p* = 0.035). Although there was a decrease in the median LDH value in the train-surfing group to 419.75 U/L, the occupational group showed relatively stable levels at 176 U/L (*p* = 0.035). The IQR remained broader in the train-surfing group (137 U/L) than in the comparison group (32.62 U/L; *p* = 0.035), reinforcing the sustained elevation and variability of LDH values over time (Figure 2).

### 3.6. Aspartate Transaminase (AST)

Train surfers consistently exhibited significantly elevated AST levels compared to patients with work-related high-voltage injuries (*p* = 0.042 for day 3, *p* = 0.035 for day 10). Within the first 72 h, the median AST level among train surfers was 346.5 U/L, while the work-related group showed a much lower median of 30 U/L (*p* = 0.042). The interquartile range (IQR) was 70 U/L in the train-surfing group versus 5.25 U/L in the occupational group (*p* = 0.042), highlighting the increased variability and severity among train surfers. Extremely elevated maximum values were found in both groups, with 2127 U/L in train surfers and 1866 U/L in the occupational cohort (*p* = 0.042).

**Figure 3:**
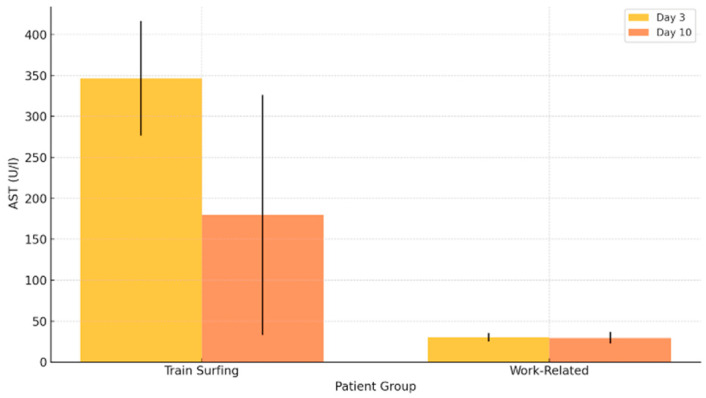
AST levels on day 3 and day 10 post-injury.

On day 10, AST levels in train surfers had declined to a median of 179.75 U/L, but remained substantially higher than the 29.5 U/L median observed in the work-related group (*p* = 0.035). The disparity in IQR also persisted (146.75 U/L vs. 7.12 U/L; *p* = 0.035), confirming the sustained systemic impact in the train-surfing cohort (Figure 3).

### 3.7. Troponin

Troponin, a key marker for myocardial injury, was markedly elevated in both patient cohorts within the first 72 h following high-voltage trauma. However, a statistically significant difference was observed between the two groups (*p* = 0.042), with train surfers showing considerably higher values. The median troponin level in the train-surfing group was 60 ng/L, compared to 10 ng/L in the work-related group. A notable divergence was also evident in the interquartile ranges, with 149.25 ng/L in train surfers versus 23.5 ng/L in occupational cases (*p* = 0.042). The most extreme values further underscored this disparity, with a peak of 1895 ng/L recorded among train surfers, while the highest value among industrial accident victims was 171 ng/L.

**Figure 4:**
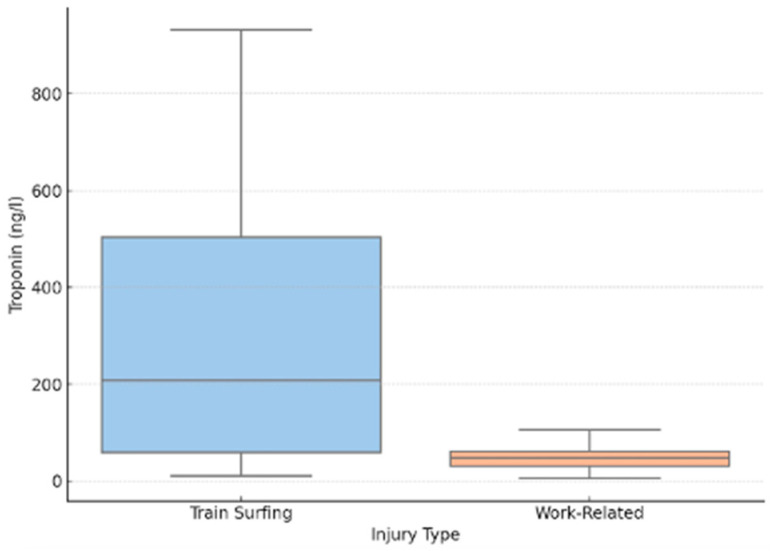
Troponin levels within 72 h post-injury.

Due to clinical routine and protocol limitations, troponin measurements were predominantly limited to the acute phase, restricting data availability to the first 72 h post-trauma. Despite this constraint, the findings clearly indicate a significantly higher degree of myocardial stress or injury in train-surfing-related high-voltage trauma compared to work-related incidents (Figure 4).

### 3.8. Myoglobin

Myoglobin levels were markedly elevated in both patient groups within the first 72 h, indicative of extensive muscle cell degeneration. However, patients from the train-surfing group consistently exhibited significantly higher values than those with work-related injuries (*p* = 0.042). The median myoglobin concentration in train surfers was 7808 ng/mL, compared to just 147.5 ng/mL in the work-related group. Additionally, the interquartile range was substantially broader in the train-surfing cohort (10,620.5 ng/mL vs. 123.87 ng/mL), reflecting greater variability and severity. Peak values also varied widely, with maximum concentrations reaching 114,800 ng/mL among train surfers and 78,320 ng/mL among occupational victims.

**Figure 5:**
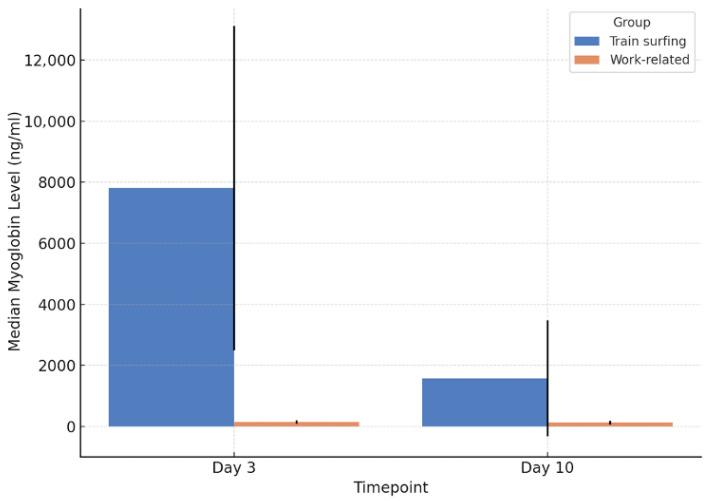
Myoglobin levels on day 3 and day 10 post-injury.

Over a 10-day observation period, myoglobin levels declined in both cohorts, yet a significant difference persisted (*p* = 0.035). The train-surfing group maintained a higher median value of 1583 ng/mL and an interquartile range of 3801.25 ng/mL, whereas the occupational group presented a median of 132 ng/mL and IQR of 130.13 ng/mL (Figure 5).

### 3.9. CK-MB

The CK-MB levels followed a similar trend to other cardiac markers, revealing significant differences between the two groups during the first three days post-trauma (*p* = 0.042). The train-surfing group showed a markedly elevated median CK-MB level of 240 U/L, compared to only 26.65 U/L in the reference group. This difference was mirrored in the interquartile ranges (128.33 U/L vs. 6.97 U/L) and maximum values (1629 U/L vs. 656.5 U/L), highlighting the severe cardiac involvement in train surfers.

**Figure 6:**
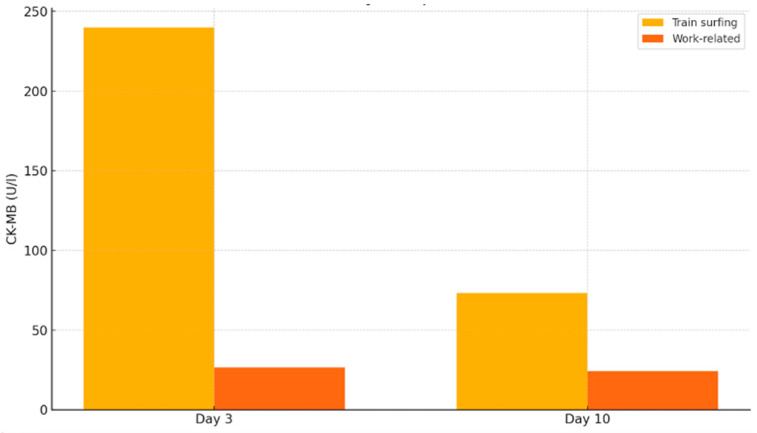
CK-MB levels on day 3 and day 10 post-injury.

By day 10, although CK-MB levels had declined in both groups, train surfers still showed significantly elevated levels, with a median of 73.3 U/L and IQR of 87.12 U/L, compared to a median of 24.25 U/L and IQR of 5.23 U/L in the occupational injury group (*p* = 0.035). These findings align with the results observed in other biomarkers and support the conclusion that train surfing is associated with significantly more extensive muscle and myocardial injury than typical work-related high-voltage trauma (Figure 6).

### 3.10. Cardiac Biomarkers and Mortality

To identify potential predictors of mortality in patients with high-voltage injuries, univariate analyses were performed for several cardiac and muscle biomarkers on day 3 and day 10 after admission. The following variables were analyzed: creatine kinase (CK), creatine kinase–MB isoenzyme (CK-MB), myoglobin, aspartate aminotransferase (AST), and lactate dehydrogenase (LDH). Patients were divided into two groups based on outcome: survivors and deceased. The tables show the number of available biomarker values for each outcome group and the corresponding *p*-values. While individual biomarker levels tended to be higher in deceased patients, no statistically significant differences were observed between survivors and non-survivors for any of the biomarkers on either day 3 or day 10. These findings suggest that although biomarker elevation is common in high-voltage trauma, isolated values of CK, CK-MB, myoglobin, AST, and LDH do not reliably distinguish between fatal and non-fatal outcomes (Table 3).

The paired comparison between day 3 and the latest available biomarker values revealed significant decreases in CK, CK-MB, and AST levels over time, suggesting a gradual resolution of acute muscle and hepatic stress in survivors. Myoglobin and LDH showed no statistically significant changes, which may reflect a more stable release pattern or limited sensitivity in this post-acute window. These results underline the utility of CK and AST as dynamic markers of injury progression or recovery in high-voltage trauma patients, while highlighting the variable behavior of different biomarkers in burn-related systemic injury (Table 4).

To further explore potential predictors of mortality, we attempted a multivariate logistic regression using the available values of cardiac biomarkers on day 3 and 10. However, this analysis could not be completed due to perfect separation—a statistical condition in which one or more predictor variables almost completely discriminate between outcome categories. In our dataset, certain combinations of highly elevated biomarker levels were exclusively observed in patients who died, while lower values were only seen in survivors. Although this precluded formal regression modeling, the presence of perfect separation paradoxically highlights a very strong association between extreme biomarker elevations and mortality.

## 4. Discussion

This study presents a comprehensive 30-year retrospective analysis of high-voltage electrical injuries, focusing specifically on cardiovascular complications and biomarker dynamics. Compared to the recent long-term study by Korkiamaki et al., which analyzed high-voltage injuries in 18 young individuals following train-surfing incidents in Finland, our findings show similar trends in terms of severity and poor outcomes [2]. In their cohort, the mean TBSA was 45%, the in-hospital mortality was 16.7%, and nearly 40% of patients exhibited late neurological or psychological sequelae. Our study likewise focused exclusively on high-voltage trauma, with a mortality rate of 20.8% among train-surfing victims, and comparably high rates of surgical interventions, ICU stays, and late complications. Both studies underline the unique vulnerability of this patient population: typically adolescent males exposed to high-voltage current in uncontrolled, dangerous environments. While Korkiamaki et al. emphasized the long-term neuropsychological burden, our analysis additionally highlights cardiac complications and elevated biomarker levels as early prognostic indicators.

Our findings also resonate with case-based reports by Jensen et al. and Kose et al., who emphasize the potential for delayed cardiac arrhythmias and conduction disturbances following electric trauma, even in the absence of early ECG changes or enzymatic signs of myocardial necrosis [23,24]. Notably, 51% of our patients showed ECG abnormalities—most within the first 24 h—including ventricular fibrillation and other malignant arrhythmias. Although our study was not designed for long-term follow-up, the significant early elevation of troponin, CK-MB, and other cardiac markers among train surfers suggests early myocardial stress that could progress to late dysfunction, as posited by Robinson and demonstrated in the prospective cardiac imaging data from Giunard et al. [25,26].

Waldmann et al. advocate for structured cardiac monitoring in high-voltage injury patients, cautioning against premature discharge due to the potential for delayed arrhythmias and systemic involvement [14,20]. Their recommendations contrast with those of Purdue, Bailey, Cunningham, and Seyfrydova, whose studies—largely based on low-voltage trauma—suggested minimal risk in asymptomatic individuals [3,27,28,29,30]. Our results, however, add weight to Waldmann’s perspective: among train surfers, we observed 25% cardiac arrest, 43.75% resuscitation, and frequent biomarker elevation, clearly justifying early intensive monitoring and individualized management strategies [14]. This distinction between high- and low-voltage injuries is further emphasized by the findings of Pilecky and Seyfrydova, whose cohorts of low-voltage injuries demonstrated negligible mortality and minimal biomarker elevation [3,21].

Furthermore, our multivariate regression identified TBSA, the need for resuscitation, and acute kidney failure as independent predictors of mortality, paralleling the findings of prior work of Koenig et al. and reflecting the interconnected pathophysiology of systemic electrical injury [4]. ECG abnormalities were not independently predictive of cardiac failure but were significantly associated with renal failure, highlighting a shared mechanism of widespread cellular injury and myocyte necrosis. The persistent elevation of biomarkers like troponin and myoglobin supports this hypothesis, as does the higher incidence of renal complications and dialysis in train surfers.

In our findings the elevation of biomarkers—such as creatine kinase, myoglobin, LDH, troponin, and CK-MB—was significantly more pronounced in train surfers, underscoring the systemic impact of vertical current flow. These findings reinforce the observations of Giunard et al., who documented persistent myocardial dysfunction long after discharge in patients with high-voltage injuries [25]. Our results also suggest that these elevations may serve not only as diagnostic tools but also as predictors of multi-organ complications, a hypothesis that should be explored in future prospective studies.

In light of these findings, we propose a differentiated approach to the management of electrical injuries. While low-voltage trauma may often be managed conservatively in asymptomatic individuals with normal ECG, high-voltage injuries—especially those involving the thorax or cranial entry points—necessitate comprehensive assessment, prolonged monitoring, and interdisciplinary care. In line with recommendations from Bailey, Giunard, and Waldmann, we advocate for at least 24–48 h of cardiac observation in high-risk patients, particularly those with vertical current flow or elevated cardiac biomarkers [14,20,25,28,29].

Our findings underscore a significantly higher burden of morbidity and mortality among train-surfing patients compared to those with work-related high-voltage trauma. This mirrors prior observations by Koenig et al., who found a markedly elevated rate of cardiac failure, resuscitation, and secondary trauma among train surfers, largely due to vertical current flow—a trajectory associated with more extensive systemic and cardiac involvement [4]. In our cohort, train surfers were significantly younger and sustained more extensive burns (mean TBSA: 49.92%) than occupational cases (mean TBSA: 17.91%), consistent with their higher ABSI scores and ICU demand. These findings underscore the unique risk profile and complex clinical course in train-surfing injuries.

Beyond clinical management, the sociobehavioral dimensions of train surfing merit attention. As Koenig et al., Lumenta, and Koller have reported, this phenomenon disproportionately affects adolescent males, often driven by thrill-seeking behavior and amplified through social media platforms [1,4,31,32,33]. In our cohort, the number of train-surfing-related high-voltage injuries remained stable over three decades, with 1–2 cases annually. However, recent years—particularly 2022 and from 2024 onward—show a rising trend in such injuries, reflecting a resurgence of this high-risk behavior among adolescents, as previously described by Koenig et al. [1]. Due to a noticeable increase in such injuries, we initiated a prevention campaign in collaboration with the Federal Railway Agency. This ongoing project includes school-based educational sessions, print and online media, and television outreach targeting adolescents aged 13 to 15. The program is carried out several times per year and involves physicians, psychologists, educators, and railway representatives working directly with students. However, reaching this group remains challenging. Social media trends play a major role in promoting risky behavior, with dedicated train-surfing fan pages and video content glamorizing the activity. In some cases, specific railway routes are even recommended for surfing. The danger of this phenomenon is illustrated by cases like that of Cuculic et al., who reported a fatal electrocution when a teenager climbed a parked train to take a selfie [10]. To address the psychosocial burden of these traumatic injuries, a self-support group was established at our clinic, connecting affected patients and families. Early feedback suggests that shared experience and psychological support are valuable components of recovery [1].

While our study focused on the early post-injury phase, the clinical relevance of elevated cardiac biomarkers remains uncertain and warrants further investigation regarding their association with long-term cardiac outcomes.

### Limitations

A major limitation of this study is the pronounced gender imbalance, with only two female patients included, which precludes any meaningful analysis of gender-specific differences in injury patterns or outcomes. Given the 30-year study period, minor changes in treatment strategies or documentation practices over time cannot be entirely excluded and may have introduced some variability in the data.

## 5. Conclusions

This study highlights the distinct and severe nature of train-surfing-related high-voltage electrical injuries compared to those sustained in occupational settings. Train-surfing victims were significantly younger, sustained more extensive burns, and experienced higher morbidity and mortality. Importantly, the train-surfing group demonstrated markedly elevated levels of cardiac biomarkers—including creatine kinase, troponin, myoglobin, and CK-MB—both within the first 72 h and up to 10 days post-trauma, indicating more profound myocardial and muscular damage. While ECG abnormalities were common in both groups, the consistently higher enzyme levels in train surfers underscore the need for heightened cardiac surveillance in these patients.

These findings suggest that train surfing constitutes a particularly dangerous and underrecognized form of high-voltage trauma, fueled in part by social media and lack of awareness about the invisible risk posed by electric arcs. Preventive efforts must therefore target younger populations through education, community outreach, and collaboration with public transportation authorities. Clinically, the data support the implementation of aggressive early diagnostics and multidisciplinary treatment strategies in suspected train-surfing injuries, particularly with regard to cardiovascular monitoring and renal protection.

Prevention campaigns targeting this demographic are essential and must be continuously adapted and reinforced. In Austria, collaborative projects with national rail services and schools have been initiated, but recurrent fatalities suggest that these efforts must be more persistent and strategic [1].

In conclusion, train-surfing-related high-voltage injuries represent a uniquely severe and multifaceted form of electrical trauma. Our data emphasize the need for targeted surveillance, advanced diagnostic strategies, and individualized treatment protocols. Future research should aim to define long-term outcomes, refine biomarker-based prognostication tools, and establish standardized management algorithms that address the full spectrum of cardiac, renal, and systemic consequences in this high-risk population.

In sum, train-surfing injuries represent a preventable, yet increasingly relevant public health issue. Greater awareness and further research are essential to reduce incidence, optimize care, and improve outcomes in this high-risk group.

## Figures and Tables

**Table 1:** Cardiac biomarkers on day 3.

Biomarker	Train Surfing (Median)	Work-Related (Median)	*p*-Value
CK (U/L)	5205.75	36.0	<0.001
LDH (U/L)	289.39	174.0	<0.001
AST (U/L)	113.47	21.75	<0.001
Troponin (ng/mL)	27.25	6.5	<0.001
Myoglobin (µg/L)	147.5	25.0	<0.001
CK-MB (U/L)	26.65	10.0	<0.001

CK = creatine kinase; LDH = lactate dehydrogenase; AST = aspartate aminotransferase; CK-MB = creatine kinase muscle-brain fraction. Units: CK, LDH, AST, and CK-MB in U/L; troponin in ng/mL; myoglobin in µg/L. Statistical significance assessed using the Mann–Whitney *U* test.

**Table 2:** Cardiac biomarkers on day 10.

Biomarker	Train Surfing (Median)	Work-Related (Median)	*p*-Value
CK (U/L)	2702.25	221.5	<0.001
LDH (U/L)	130.13	146.88	<0.001
AST (U/L)	71.34	26.88	<0.001
Troponin (ng/mL)	7.12	5.23	<0.001
Myoglobin (µg/L)	1728.1	132.0	<0.001
CK-MB (U/L)	27.65	27.65	<0.001

CK = creatine kinase; LDH = lactate dehydrogenase; AST = aspartate aminotransferase; CK-MB = creatine kinase muscle-brain fraction. Units: CK, LDH, AST, and CK-MB in U/L; troponin in ng/mL; myoglobin in µg/L. Statistical significance assessed using the Mann–Whitney *U* test.

**Table 3:** Univariate analysis of laboratory biomarkers in relation to patient mortality on day 3 and day 10 following high-voltage injury. Group comparisons between survivors and deceased patients were performed using the Mann–Whitney *U* test, as most variables were non-normally distributed. No statistically significant differences were found. Legend: CK = creatine kinase; CK-MB = creatine kinase–MB isoenzyme; AST = aspartate aminotransferase; LDH = lactate dehydrogenase.

	*p*-Value	Survivors (n)	Deceased (n)
CK (Day 3) (UM	0.3776	43.0	6.0
Myoglobin (Day 3) [ng/mL)	0.789	28.0	5.0
CK-MB (Day 3) [UM	0.5897	36.0	6.0
AST (Day 3) [U/L]	0.4735	43.0	6.0
LDH (Day 3) [U/L]	0.4735	43.0	6.0
CK (Day 10) [U/L]	0.8611	25.0	6.0
Myoglobin (Day 10) [ng/mL]	0.456	11.0	3.0
CK-MB (Day 10) [U/L]	0.4494	14.0	3.0
AST (Day 10) [U/L]	0.5753	27.0	6.0
LDH (Day 10) [U/L]	0.9064	26.0	6.0

**Table 4:** Comparison of cardiac and muscle injury biomarkers at day 3 versus day 10.

	Wilcoxon *p*-Value
CK (U/L)	<0.0001
Myoglobin (ng/mL)	n.s.
CK-MB (U/L)	0.0015
AST (U/L)	0.0067
LDH (U/L)	n.s.

## Data Availability

Data are available upon request.
